# Diversity in patient and public involvement in healthcare research and education—Realising the potential

**DOI:** 10.1111/hex.13896

**Published:** 2023-10-23

**Authors:** Sarah Hatch, Jim Fitzgibbon, Amanda Jayne Tonks, Liz Forty

**Affiliations:** ^1^ Public Involvement and Engagement Team, School of Medicine Cardiff University Cardiff UK; ^2^ Centre for Medical Education, School of Medicine Cardiff University Cardiff UK

**Keywords:** diverse patient/public, health research and education, involvement

## Abstract

**Background:**

Patient and public involvement (PPI) is an increasing priority in health‐related research and education. Attracting and supporting people from different demographic groups to give up their time and get involved is important to help ensure that all parts of society are empowered, represented and their voices heard in decisions that may affect their health and quality of life.

**Objectives:**

(1) To determine if a demographically diverse cross‐section of society would be interested in contributing to healthcare research and education. (2) To understand factors that can act as barriers and enablers to effective and diverse PPI.

**Method:**

PPI survey data was collected via engagement events, with the aim of scoping interest in PPI from a diverse public. A Focus Group study involving members of the public, academic and professional service staff, was then conducted to gain a deeper understanding around the barriers and enablers of diversity within PPI.

**Results:**

71% of a diverse rich public indicated they would like to get involved in healthcare research and teaching. 76% of survey respondents indicated that they would be happy to share a personal or family experience of healthcare. The two biggest factors impacting on our cohort getting involved are’ availability of time’ and ‘being aware of PPI opportunities’. These factors may disproportionally affect specific groups. Shared and individual PPI enablers and barriers were identified across all stakeholder groups within the Focus Group Study, as well as generic and novel factors that would impact on an institutions’ ability to improve PPI diversity.

**Conclusion:**

These data points confirm a demographically diverse public's appetite to get involved in academic health research and teaching. This needs to be recognised and harnessed to ensure public contributor networks are representative of society. Equality Impact Assessments should be undertaken in relation to all PPI opportunities. There is a need to recognise the investment of time and resources required to build mutually beneficial relationships with diverse communities as well as the development of inclusive ‘fit for purpose’ PPI infrastructures to support the uptake of diverse PPI contributors.

**Public Contribution:**

This study involved members of the public responding to a short survey. Public contributors made up one of the three focus groups. The School of Medicine lead public contributor was also involved in the preparation of this manuscript.

## 1 INTRODUCTION

Patient and public involvement (PPI) in healthcare education and research is an increasing priority for several stakeholders including regulators,^1^, ^2^, ^3^ research policymakers^4^, ^5^, ^6^, ^7^; research funders^8^, ^9^; and charities.^10^ Many see PPI as essential to ensuring relevance and meeting societal needs. However, public contributor diversity remains a challenge, as does genuine partnership work to ensure PPI influences and makes a difference to higher education research and teaching.

The term ‘patient and public involvement’ has traditionally been used to describe the involvement of patients and members of the public in strategic decisions around health services and policy. Increasingly, PPI has become valued in healthcare research, education and training, where there is a broad spectrum of involvement between patients and the wider public. Public contributor involvement may, for example, refer to patients helping to shape a particular health service, members of the public informing a research proposal, or patients and/or family members delivering training to healthcare students. Although patients are also members of the public, the term ‘patients’ in addition to the term ‘public’ is used here, as in the context of healthcare research and education, ‘patients’ have a specific role to play.

Diversity of individuals from a broad range of backgrounds is crucial to ensure results of health and social care research are more widely applicable, and that teaching/training in health and social care meets the needs of the whole population.

Patients have long been a part of healthcare education. In the past, it was generally assumed that patients would support the delivery of healthcare education. However, the increased focus on more equal partnerships in terms of shared decision‐making in clinical practice and patient‐centred care has highlighted a need for different approaches to involving patients in the education of healthcare professionals. Increasingly within healthcare education, a more structured approach to patient involvement is being taken, which does not rely solely on opportunistic patient contact in clinics and wards. There continues to be significant variation in the extent of, and quality of, PPI in healthcare education, with specific groups of individuals being more likely to become involved.^11^, ^12^

PPI in research is where research is ‘being carried out “with” or “by” members of the public not just “to”, “about” or “for” them’.^13^ This definition can also be applied to patient involvement in teaching where, in line with patient‐centred care and shared decision‐making, educational experiences require that the patient is actively involved in the learner experience. Here patients and the public contribute as experience‐based experts whose knowledge is complementary to that of clinicians and academics.^14^

Patients and the wider public can be involved in different aspects of teaching and research, and this can have several benefits for all involved, including: Members of the public feeling empowered and valued, gaining confidence and life skills; researchers developing a ‘greater understanding and insight into their research area, gaining respect and a good rapport with the community’; the community becoming ‘more aware and knowledgeable about their condition’.^15^, ^16^, ^17^, ^18^

Despite progress in the field, PPI continues to be open to criticisms of being tokenistic, selective and unrepresentative.^19^ Often where PPI is implemented, it is on a local individual level, for example, reviewing researcher lay summaries and patient information sheets or sharing personal experiences with healthcare students. There is limited evidence of PPI on a more strategic level^20^ and although PPI has significant short‐term benefits for all involved, there has been little research into the longer‐term benefits.^21^

In 2017, an exercise recording instances of PPI taking place across Cardiff University School of Medicine highlighted:

1. PPI requires human resources to manage a variety of practical matters, including PPI recruitment (of diverse and under‐served communities), induction, training, support, communication and recognition.
2. The ‘Publics’ already involved tend to be ‘white’, ‘middle‐class’ ‘highly educated’, ‘retired professionals’ and are often involved in several PPI projects across the region.^22^
3. An absence of identified dedicated PPI support can create inconsistencies in some issues which affect the PPI experience (including prompt PPI payments; communicating feedback; impact of PPI).

There is a growing resource for published work, particularly in the PPI in research field that provide a guide as to what good PPI looks like.^23^, ^24^ Similarly, in education, Le Var advocates for a strategic approach to make PPI an effective and workable reality, although there has been little advance in this direction.^25^ A plethora of worldwide frameworks supporting PPI in research now exist. However, Greenhalgh et al^26^ questioned the utility of the usefulness of a single off the shelf framework and instead suggested an adaptable and flexible ‘menu of evidence‐based resources which stakeholders can use to co‐design their own frameworks’.

Across Higher Education Institutions (HEIs), coordinated and sustained programmes of PPI in research and education are rare. These require an investment commitment to enable involvement, with diverse patient and community groups that is meaningful for all involved, but that is also practical and well supported. To obtain senior level institutional buy‐in, there is a recognised need for the systematic evaluation and dissemination of the outcomes of PPI across research and teaching, to strengthen the evidence base around PPI and improve our understanding of what effective and inclusive PPI looks like.^27^

We ran an evaluation survey with members of the public to engage a diverse public and explore barriers and enablers to involvement in healthcare research and education at Cardiff University.

We then ran a Focus Group Study with the aim of obtaining a deeper understanding of the factors that can act as barriers and enablers to effective and inclusive PPI in a university setting.

PPI was defined as:

teaching and/or research related activities that are carried out with or by members of the public or patients, not to, about or for them.^13^

## 2 METHOD

### 2.1 Public Engagement Events: Getting involved in healthcare research and education within the University setting

We ran three events to highlight to the public the opportunities that are available to them to get involved in healthcare research and teaching within the University setting. We explained that our overall aim was to increase diversity within PPI, and the first stage of this was to go out into the community to engage with the public about PPI opportunities.

As part of these events, members of the public could fill in an evaluation survey (see Supporting Information: Appendix 1), asking them about their views and experiences of PPI and whether they are/would like to be involved. Members of the Public were able to include their demographic details if they wished to for the purposes of evaluating diversity in the Publics we accessed and were also able to include their personal details if they wanted to get involved in University PPI. Members of the public were advised to leave blank anything that they preferred not to complete.

#### 2.1.1 Format of events

The events were run at two Cardiff locations and one neighbouring county location in the Vale of Glamorgan. Cardiff's population is recognised to be diverse. According to the 2021 census figures Cardiff had the highest proportions of people identifying within the high‐level categories ‘Asian, Asian British or Asian Welsh’ (9.7%), ‘Black, Black British, Black Welsh, Caribbean or African’ (3.8%), ‘Mixed or Multiple ethnic groups’ (4.0%) and ‘Other ethnic group’ (3.3%). Two of the events were held within high footfall areas of two university teaching hospitals: University Hospital of Wales (Cardiff) and the University Hospital of Wales Llandough (Vale of Glamorgan) https://cavuhb.nhs.wales/, as well as an early evening family science engagement event held in the local science discovery centre (https://www.techniquest.org/).

Members of the public approached a stand with free ‘Get Involved’ merchandise and were invited to complete the evaluation survey (we did not actively approach the public). Respondents were provided with information on the project and how data would be used, stored, shared and consent obtained. At Techniquest, the ‘Get Involved’ survey stand was part of a stamp collection initiative resulting in a free prize for all completed stamp cards.

At each event, views on payment for involvement (Should public contributors be paid for their involvement?) were sought utilising a token tube collector tool. There were four options to choose; (i) no payment; (ii) expenses and travel; (iii) vouchers and (iv) hourly rate. Each respondent was able to select only one option.

### 2.2 Focus group study

#### 2.2.1 Design

Focus groups were conducted to gain a deeper understanding around the barriers and enablers for diversity and inclusivity in PPI within the University setting. This qualitative study followed a constructivist approach with the aim of identifying the social, cultural and structural contexts that influence an individual's experience.

#### 2.2.2 Participant recruitment

We contacted individuals who were currently involved in PPI activities across the College of Biomedical and Life Sciences at Cardiff University. These included university staff and members of the public. All participants were aged 18 or over at the time of the study and had the capacity to provide written informed consent.

Potential participants (those who had expressed an interest or were recently involved in PPI activities) were sent an email or letter to invite them to participate in a focus group study with the aim of improving our understanding around what motivates patients and the public to get involved in university research and education, to inform the development of an inclusive patient and public recruitment strategy and increase diversity within PPI. The communication also included a copy of the participant information sheet and consent form. Reminder invitations were sent one week after the original email to increase recruitment rates and ensure that all who would like to take part had the opportunity to do so.

Three separate groups of four participants were formed to allow group members to feel comfortable expressing their views and experiences; (i) members of the public/patients, (ii) academic staff and (iii) professional service staff.

The members of the public group all had experience of involvement in research and/or teaching at a HEI. The professional services staff all had experience in facilitating public involvement in research and/or higher education teaching and assessments. The academic grouping consisted of staff on fixed term and tenured contracts with experience of embedding PPI within their own areas of research and teaching.

#### 2.2.3 Data collection

The focus group instruments were designed following an inductive approach, reflecting the key focus of this study but being open to allow the participants to guide the content of the focus group. The instruments included questions around personal drivers and barriers to involvement, recognition for involvement and diversity within PPI, and views on a draft PPI consultation document.

Potential participants were provided with a participant information sheet a week before the focus group and offered the opportunity to discuss the project with the researchers. They were then asked to give written informed consent. When participants completed the consent form, they could choose to give consent for their anonymous quotes to be used in written reports and publication. Participants were informed that this was optional, and that if they chose not to consent to quote use, they could still take part in the focus group. No participants requested that their quotes were not to be used.

Focus groups met for approximately 2 h each and were moderated by two researchers (who also took brief notes during the focus groups). Focus groups were audio recorded and professionally transcribed. The audio files and written notes were used by the authors to check the accuracy of the transcription. The data and materials from the meeting were anonymised.

All data and materials (including the digital tape recordings) were stored and processed securely in line with university guidelines and the Data Protection Act. Electronic data were stored on password protected computers. All identifiable information was stored separately to focus group data. Data will be stored for 5 years after study completion in line with university guidelines. The study received ethical approval from Cardiff University School of Medicine.

#### 2.2.4 Validity and reliability

During the focus groups, summaries of main discussion points were used to determine accuracy. Participant review of the report occurred at the conclusion of the project, to ensure the authenticity of the work.

### 2.3 Data analyses

#### 2.3.1 Survey data

All survey forms were included. Questions left blank were counted in the ‘Not specified’ category.

Descriptive data are presented from the public survey. The number (*n* = ) of respondents answering each survey question are included in the relevant figure title.

In total, 118 survey responses were collated from members of the public on involvement in university activity. 112 members of the public responded to the Token Tool Collector question ‘Should public contributors be paid for their involvement?’

#### 2.3.2 Focus group data

A thematic analysis of the focus group data was conducted following an inductive approach, whereby data were open‐coded and participant/data‐based meanings were elicited. The transcripts were read multiple times to ensure familiarisation with the data. Each group transcript was independently reviewed and then iteratively coded by two researchers per focus group. When rereading the data generated no new meanings or patterns, semantic codes were created. The paired researchers then met to discuss and agree on the emergent themes identified for each group. Thematic patterns were recorded according to the topics that were discussed in depth and length by the groups. The authors (S. H.; A. T.; L. F.) reviewed themes and analysed the data to determine common and divergent themes across the three groups. The results are described using quotations as illustration.

## 3 RESULTS

### 3.1 Public survey

The 118 participants who completed the survey represented a richly diverse representation of the Cardiff population. Demographic data is presented in Table 1. Ten individuals did not complete the demographic data section. The majority of the individuals who completed the survey were between 25 and 64 years of age (70%).

**Table 1:** Demographic data of public survey respondents and those interested in getting involved in patient and public involvement activity.

	% of total survey participants	% of participants interested in getting involved
*Age range of participants*		
25–44	39	41
45–64	31	36
65–74	8	6
18–24	6	7
75+	3	2
Prefer not to say	2	1
Not specified	11	7
*Sex of participants*		
Female	63	69
Male	25	22
In another way	1	0
Prefer not to say	1	1
Not specified	10	8
*Sexual orientation of participants*		
Heterosexual/straight	72	75
Bisexual	2	3
Other	2	1
Gay Man	2	2
Gay Woman	2	2
Prefer not to say	8	6
Not specified	12	11
*Participants identifying with a disability*		
No disability	66	69
One or more disability identified	20	18
Prefer not to say	2	0
Not specified	12	13
*Religion and belief*		
No religion	38	41
Christian	28	27
Muslim	7	7
Any other religion or belief	4	4
Spiritual	2	1
Buddhist	1	1
Hindu	1	0
Atheist	1	1
Prefer not to say	7	7
Not specified	11	11
*Ethnicity*		
White	71	75
Other Asian background	3	5
Asian or Asian British—Indian	2	1
Mixed‐White and Black Caribbean	3	4
Black or Black British—Caribbean	2	2
Asian or Asian British—Bangladesh	2	0
Black or Black British—African	1	0
Asian or Asian British—Pakistani	1	2
Chinese	1	0
Arab	1	1
Prefer not to say	2	2
Not specified	11	8

The ethnic diversity of the cohort was representative and some of the high‐level categories were similar to that of the local authority area, namely our Asian categories total 8% compared with 9.7%; our Black categories total 3% compared with 3.8% and our White category is 71% compared with 79.2% (Office of National Statistics, 2021).^28^ 18% of those interested in getting involved; 36% of those already involved; and 22% of those not interested in getting involved, identified themselves as having one or more disabilities. The list of disabilities included in the survey can be found in Supporting Information: Appendix 1.

71% of our survey respondents indicated that they would like to get involved in university research and teaching in some way, with 9% saying they were already involved. The majority of those interested in getting involved were between the ages of 25 and 64. 75% described their ethnic group as white; a total of 8% identified belonging in the high‐level Asian categories; and 4% identified in the ‘Mixed’ category. These figures aligned closely (79.2%; 9.7% and 4%, respectively) to recorded ethnic diversity figures for Cardiff (Office of National Statistics, 2021).^28^ 41% of the interested cohort indicated ‘No religion’ reflecting 2021 census data (where 42.9% reported having ‘No religion’, making it the most common response in the Cardiff local authority area).

**Figure 1:**
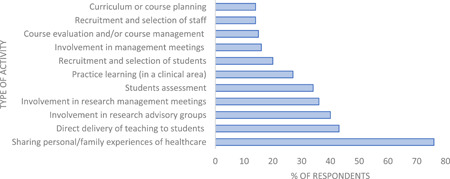
Activities of interest to survey respondents interested in patient and public involvement (*n* = 97).

As can be seen in Figure 1, when asked about their interest in being involved in specific PPI activities, ‘the sharing of personal/family experiences of a clinical problem’, was the most frequently rated activity by respondents. Being involved in the direct teaching and assessment of students and participating in research management and advisory group meetings were the next popular activities of interest amongst our cohort.

**Figure 2:**
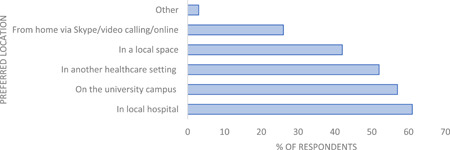
Survey respondents preferred location for patient and public involvement activities (*n* = 99).

When asked about their preferred location for carrying out PPI activities (see Figure 2), respondents rated hospital/university settings and local spaces as preferable locations, with 26% of respondents saying they would like to engage with PPI virtually (e.g., prerecorded online materials, live webinars/online meetings, etc.)

**Figure 3:**
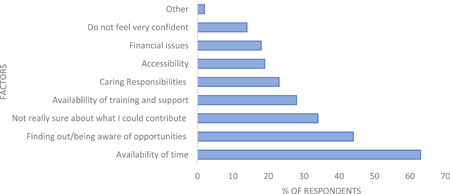
Survey responses of factors that most impact on an individual's ability to engage with patient and public involvement activity (*n* = 104).

When asked about the factors that had the most impact on an individual being able to get involved in PPI activities, the two factors rated most frequently by respondents were (i) ‘availability of time’ and (ii) ‘finding out/being aware of PPI opportunities’ (see Figure 3). Not being sure about what they could contribute; availability of training and support and caring responsibilities were the next group of factors impacting our public survey respondents. Survey respondents who indicated that they would not be interested in getting involved highlighted the following reasons for their decision: lack of time (family; work and health commitments); no transport; reside outside of area; lack of confidence; not sure what it would involve and not feeling comfortable.

**Figure 4:**
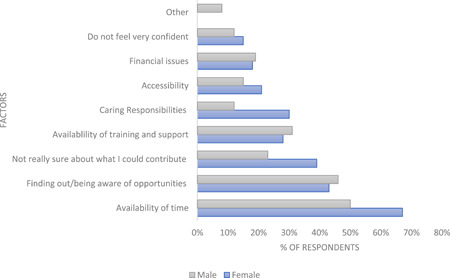
A breakdown of male and female responses to factors impacting the ability to get involved in patient and public involvement activity (*n* = 67 females; 26 males).

An analysis of female and male responses to factors that impact on getting involved in PPI activities is highlighted in Figure 4. This illustrates that the availability of time; not being sure about what they could contribute and caring responsibilities impact more on our female respondents than our male respondents.

In response to being asked whether they would like to receive payment for their involvement in University PPI activity, 48% of respondents stated that they would like their expenses and travel to be reimbursed by the university; 27% said that they would prefer not to receive payment; 17% confirmed they would appreciate thank you’ vouchers in recognition of their contribution and 8% of respondents said they would like to receive an hourly rate.

### 3.2 Focus groups

Focus group analysis identified several themes and highlighted a variety of opinions in respect of a support infrastructure for PPI with the aim of increasing PPI diversity. The first theme focused on Enablers and Barriers to PPI and included several sub‐themes. These were Definitions/Clarity of Roles, Training, PPI as a Profession, and Payment. The second theme, Central and Local PPI support, focused on the shared elements of PPI support across an institutional setting, along with the elements that are specific to a particular project or subject area. The final theme focused on Diversity. The main themes discussed are further explored in Table 2.

**Table 2:** Exploration of discussed themes raised within each focus group.

**Theme 1**	**Enablers and barriers to PPI**
**Focus group**	**Definition/Clarity of roles within PPI: Discussion areas of focus and relevant quotes**
All	Agreed definitions, a clear understanding of the role that PPI can play in adding value to health research and teaching as well as clarity on the mutual expectations of all stakeholders involved will help to support members from all corners of society to get involved in PPI activity.
	Both the professional service staff group and the academic group highlighted the term ‘involvement’ may not be appropriate for all instances within teaching particularly within assessments where the role is passive but still essential and a requirement. It was agreed that clear categories of ‘involvement’ within teaching do exist and should be used to clarify ‘public involvement in teaching’ including: design and delivery of curriculum; student selection processes; programme validations; and fitness to practice panels.
Academic	Embed Equality, Diversity, and Inclusion (EDI) within PPI roles and descriptors with a focus on providing a ‘patient/public perspective’.^29^, ^30^ It should not include aspects of an academic researcher or lecturers work, despite this being advocated as valid PPI activity in some areas.^31^ ‘***Normally the thing that public contributors get asked to do is a research interview and analyse the data. I studied ten years to do this well. It is not fair and should not be asked of a patient/public contributor involved in research***’.
	Roles and expectations of both public contributors and academic staff need to be managed appropriately so all contributions are valued and expertise acknowledged. (UK Standards for Public Involvement [Working Together])^23^
Public/patient contributor	Whatever the PPI role, it is important that PPI boundaries are defined and roles and responsibilities are detailed at the outset. ‘***Why do you want me? What can I offer? No matter how big or small***’. (UK Standards for Public Involvement (Working Together)).^23^
	**Training: Discussion areas of focus and relevant quotes**
Public/patient contributor/Academic	Optimising PPI experiences to attract and encourage diverse individuals to get involved. It is important that PPI contributors have an induction and can access tailored training that complements existing general training programmes run by national bodies such as Health and Care Research Wales.
Academic	The provision of support and academic mentoring on PPI best practice is an unmet need. PPI support infrastructures need to offer flexibility to enable individual academics to decide which method meets the needs of their research and/or teaching field. It is important ‘**we are able to learn** ***from our own mistakes***’ and can develop new ways to involve patients and the public.
	Limited and different PPI banks working in isolation can sometimes lead to public contributors operating in different parts of the country. PPI contributors who serve on multiple groups, raises concerns around conflict of interest and potential breaches in confidentiality.
	‘***I hear people criticising research groups from other areas… That's not right***’.
	It is important PPI contributors receive appropriate training for the roles that they are being asked to perform (UK Standards for Public Involvement [Support and Learning])^23^ For PPI in research roles, understanding research integrity principles, for example, potential conflict of interest and confidentiality issues is important, particularly where the PPI contributor is involved in more than one research project.
	**Professionalisation of the PPI role: Discussion areas of focus and relevant quotes**
Academic	The limited pool of PPI contributors supporting healthcare research and education raises concern on the professionalisation of the PPI role.
	‘***I think that there are some professional PPI people… whom get involved in more than one project …Hitting two meetings per afternoon…***’ The PPI contributor role should not be seen as a job, ‘***it is not a job, you don't get promoted*** ‘.
	‘***I've noticed that public contributors that I'm working with have changed a lot…I think contributors should not be working for longer than a certain period of time, because I think that some become very influential …things happen with egos, competitiveness and how they treat researchers***’.
	There is concern that PPI Contributors who find themselves indefinitely getting involved in committees ‘***become institutionalised***’. Similarly in teaching situations, repeatedly asking the same PPI contributor to share their own personal healthcare experience because it is easier than recruiting a new PPI contributor can be detrimental if no longer relevant or valid because of healthcare advancements. In such situations, PPI contributor roles should come with a specified length of tenure.
	Conversely, it is over time that PPI contributors can grow in confidence and build their involvement experience in support of health research and teaching. ‘***One of my public contributors is holding £8 M of funding as a co‐applicant across various projects and is named on sixteen papers***’. Clearly this can also have positive implications for research and teaching.
	With the publication of the UK Standards for Public Involvement,^23^ there is early concern that some members of the public from diverse demographic backgrounds will be put off and discouraged from getting involved due to all the requirements expected within the standards. Guidance that aims to support and facilitate involvement ‘***becomes such a nightmare in terms of administration … and a barrier to spontaneous involvement in decision making or influencing***’. Ad hoc potentially diverse PPI will become less likely.
	**Payment: Discussion areas of focus and relevant quotes**
All	Reimbursing expenses and paying (whether financially or via other means) PPI contributors in a timely and efficient manner will help support the recruitment of public contributors from diverse demographic backgrounds.
Academic	The payment of public contributors is now universally accepted to show value for time given up to support research and teaching. Standard payments (based on guidance^32^, ^33^) should be offered in return for time given up.
	Currently, the process to reclaim expenses includes completing a form which is not specifically tailored to PPI contributors and traditionally did not allow for BACS payment. The process ‘***is quite lengthy in terms of public contributors having to wait a couple of months to receive payment for their travelling expenses and filling out the form***’. Out‐of‐pocket travelling and accommodation expenses should ideally be paid up front by the institution wherever possible. As institutions have become cashless in recent years, ‘time banking models’ and offering vouchers are growing in popularity to avoid the exchange of money.
	Traditionally, some teams within organisations (such as Universities and NHS Trusts) have experienced difficulty paying honoraria due to the way PPI activity has been classified as falling within self‐employed status. Continued efforts are being made to clarify this position with HMRC.^34^
Professional services staff	To date, the University increasingly relies on the goodwill of public contributors who often experience unreasonable lengthy delays for the reimbursement of ‘out of pocket’ expenses. The design of special provisions for this growing number of valued contributors will help the university to avoid potential risks of ‘bad feeling’ amongst our PPI contributors and strengthen our success rate in applying for research funding where PPI is a requirement.
	The adoption of standardised payment options^35^, ^36^ would eradicate payment discrepancies that currently exist across the sector and ensure that PPI is appropriately costed and budgeted for.
Public/patient contributor	‘***I struggle with the expense form and I've been doing it for three years…payment takes six weeks to two months to come through***’. (UK Standards for Public Involvement (Inclusive Opportunities)).^23^
	Organisations should book travel for PPI contributors in advance, wherever possible or provide ‘out of pocket’ expenses on the day incurred. Standard payment options for different categories of roles across healthcare research and teaching across the sector should be created. If no tailored PPI contributor claim forms are forthcoming, university staff should complete the required forms alongside the PPI contributor on the day of the PPI activity and send to relevant finance colleagues to speed up the process.

**Theme 2**	**Central and local support for PPI activity: Discussion areas of focus and relevant quotes**
All	All groups agreed the existence of a central set of institutional principles to support inclusive PPI activity across research and teaching activity would be beneficial to all stakeholders involved and help to facilitate diversity amongst PPI contributors.
Public/patient contributor	To ensure individual needs of diverse PPI contributors are met, it is important to have a recognised PPI pathway within organisations/academic institutions that is recognised within institutional support services such as finance and HR to ensure consistent and prompt payment for time and out‐of‐pocket expenses and other costs involved such as the payment of childcare costs.
	Institutions should offer opportunities for PPI contributors to share experiences, providing a sense of fulfilment in giving back to the community and to network with other PPI contributors and University staff.
Professional services staff	Colleagues from teaching and assessment teams saw the benefits in establishing central PPI support including a database of PPI contributors that could be maximised across healthcare research/teaching and assessment teams due to the constant struggles to recruit enough members of the public to support the assessment of undergraduate healthcare students. Colleagues from research teams with established local PPI structures raised concerns of potential additional bureaucracy when accessing a centrally maintained database and highlighted the differences required for involvement in research and teaching and participation in assessments: ‘***I think involvement and research is a bit more bespoke and is more disease specific;*** ‘ ‘***This structure needs to be built from the ground up***’.
	The following points of a central PPI function were unanimously supported:
	Providing an ‘***overall picture of what an organisation/institution is doing and who is doing what***’ in respect of research and teaching involvement and assessment participation.
	Providing clear definitions and expectations of research and teaching involvement and assessment participation.
	Creating an online PPI Directory of Expertise to include the contact names of staff already recruiting members of the public and detail all PPI groups (disease/School/Centre/Unit specific) that already exist throughout an institution. As well as contacts of national charitable organisations that could help to recruit patients with specific conditions.
	Delivering EDI corporate support and effective implementation of the six UK Standards for PI^23^ across an organisation.
	‘***Collating data of the inputs and outputs***’ of involvement and evaluating and measuring the impact for internal and external stakeholders.
	Developing ‘adaptable’ and ‘flexible’ best practice processes and procedures, learning from the experience of established PPI groups internally and externally, ‘making it easier’ for academics new to PPI.
	Developing and coordinating bespoke training, utilising and complimenting existing training taking place internally and externally.
	Alongside the presence of a central support function, individual specialist leads within research, teaching and assessments would be identified across an institution. Establishing excellent working relations with existing PPI groups and colleagues across an institution will maximise existing PPI resource and ensure that all requests for PPI were prioritised and met within agreed timescales. The interaction and separation of function between a central and local PPI teams could potentially benefit and learn from the centralisation of some other key functions across large institutions such as timetabling.
Academic	The need to embed diverse PPI activity within the core culture of institutions was seen as essential. A dedicated resource to help facilitate PPI activity, similar to knowledge transfer would be a valuable asset within universities. Having a support infrastructure in place and best practise models alongside a dedicated team would help to embed meaningful and diverse PPI within an institution.
	Some organisations may say ‘***that they support PPI…as they understand it is good for business. However, making a public show of supporting PPI is not the same as supporting it*** ‘. ‘***There needs to be more evidence and understanding of the benefits of PPI to support long term investment in it***’.
	The following points of a central PPI support function and database were noted:
	Several databanks of publics exist internally and externally to institutions each requiring identified dedicated resource to maintain, develop and target PPI opportunities across the database. A function of central PPI support would be to understand where public involvement databases already exist across an institution and externally and to sign post to these where appropriate. Importantly, the efforts of central support could then focus on recruitment strategies attracting diverse, and underserved groups.
	The administration involved in facilitating and evidencing the added value of PPI (e.g., reporting on the impact of PPI activity (UK Standards for Public Involvement: Impact)^23^ takes time to do well. Evidencing the value of PPI to all key stakeholders including academic staff/project teams/institutions/patients and public and students is an important identified facilitating central support function. The development of such PPI case studies would help to raise the profile of PPI activity and its value‐added benefit as well as build a strong evidence base of PPI impact (positive as well as negative) on research and teaching activity.

**Theme 3**	**Diversity: Discussion areas of focus and relevant quotes**
All	Attracting diverse PPI contributors to get involved in university research and teaching was recognised as being important to all three focus groups, particularly as PPI continues to grow in priority. Two barriers highlighted by our public survey were also echoed within the focus groups namely, ‘a lack of awareness of university PPI opportunities amongst the public’ and ‘time available’. Currently a very select group of individuals are involved in PPI as a result of members of the public generally not being aware of PPI opportunities. The availability of time to do PPI activity also impacts on both staff (academic and professional service) and PPI contributors themselves.
Public/patient contributor	Investing in raising the profile of why and how patients and members of the public can play a valuable role in supporting the core functions (research and teaching) of universities.
	‘***The need to get out into the community*** **…**’ to encourage a more diverse cross‐section of the population to get involved in university activity (e.g., GP surgeries; knitting clubs; social media community groups; libraries; Commissioners Officers in Wales; Charities; Wales Council for Voluntary Action; FE Colleges) to counter typical PPI contributor (male, retired) and natural attrition rates.
Professional services staff	‘***Trying to create a diverse, inclusive group that can do any type of involvement… without a lot of money and time is impossible***’. A significant amount of time and resource is required to build mutually beneficial public contributor relationships across diverse communities. Sustainable long‐term investment in resource as part of an institution's infrastructure could play a valuable role in the following ways:
	Recruiting PPI contributors based on priority needs at any given time. Having oversight of all PPI activity across an organisation to ensure appropriate sign‐up to a central database and/or effective signposting to established PPI groups and/or relevant identified leads.
	Establishing effective community links working with an institution's strategic partners.
	Working with different communities to ensure the EDI aspects of PPI opportunities, being creative and innovative in the establishment of an inclusive PPI database.
	Working with internal and external stakeholders to remove potential PPI barriers that exist internally within an institution and externally across society.
	Ensuring the experience of each public contributor getting involved in university activity is of the highest quality, ensuring positive repeated involvement from PPI contributors.
	Understanding the needs, wishes and ways of every member of the public wanting to get involved in the work of a university.
	Accessing communications and marketing expertise to raise the profile of PPI and ensure ‘signed up’ PPI contributors receive targeted communications via several digital and non‐digital routes.
Academic	‘***The public contributors we tend to attract are educated patients who are well informed, they know the healthcare system and their illness. Diversity is an issue…particularly when it's a university… a lot of people are going to be alienated by that***’.

*Note*: Direct quotes are in bold, italics. Areas of discussion that now form part of the UK Standards for Public Involvement have been identified and included in parenthesis.

Abbreviation: PPI, patient and public involvement.

## 4 DISCUSSION

### 4.1 Summary of principal findings

Encouraging a more diverse public to get involved in healthcare research and teaching is a growing priority within the PPI field, amongst healthcare research and education focused institutions as well as regulators such as the General Medical Council and Nursing and Midwifery Council. We found that there is interest from a diverse public to get involved in academic research and teaching with 76% of our public survey respondents indicating that they would be happy to share a personal or family experience of healthcare. This provides an insight into members of the public's willingness to share information if it is seen to be helpful to future healthcare research and education.

The study identified several factors that can impact PPI generally and effectively act as a barrier to achieving diversity in PPI within a university healthcare research and education setting. These include:

1. Breaking down the ‘ivory tower’ perception of university settings.
2. Ensuring PPI inclusivity.
3. The role of the PPI professional and support infrastructure.

#### 4.1.1 Breaking down the ‘ivory tower’ perception^37^, ^38^ and getting out into the community

Both the survey and focus group study affirm the importance of investing in ways to get out into the community,^39^, ^40^ to get to know and build relationships with different groups to support PPI recruitment. Mutual trust and respect needs to be established to overcome initial feelings of alienation and discomfort that can arise when working with a university as a PPI contributor. Understanding the needs and expectations of each member of the public wanting to get involved will support the building of sustainable relationships with diverse communities and help facilitate mutually beneficial PPI relationships for all involved.

The study affirms the importance of building an inclusive, diverse and responsive database of patients and public willing to support university led healthcare research and teaching needs. Currently, researchers can access established small self‐selecting networks of PPI contributors. These need to be expanded to be more representative of society. A collective and collaborative effort between all relevant organisations to reach out to the community to attract diverse PPI contributors will reap significant advantages, not only to HE institutions but to all other relevant stakeholders.

Expanding such networks will help to reduce the burden on the small pool of existing self‐selecting PPI contributors and help mitigate against several concerns/sensitivities raised in this study surrounding the ‘power base’ that can potentially be built up by PPI contributors involved in research, often referred to as ‘Professional’ PPI Contributors. In a study^41^ of researchers’ attitudes towards PPI in health research, the ‘professionalised’ PPI member was regarded by researchers as lacking authenticity. Thompson et al.^42^ indicate that becoming ‘professionalised’ poses dilemmas, since it involves a loss of ‘freshness’ and a loss presumably, of experiential expertise.

Developing more representative PPI databases and implementing EDI focused PPI support, policies and processes, for example, co‐defining the remit of the PPI role (different roles for different levels of experience), considering length of tenure and conflicts of interest, alongside Equality Impact Assessments, will enable the development of inclusive PPI with broader representation from across society.

#### 4.1.2 Ensuring PPI inclusivity

Our work illustrates two challenges that significantly impact on PPI diversity and inclusivity, namely ‘lack of time’ and a general ‘lack of understanding about what PPI in university activity means’. Whilst these two challenges are relevant to PPI more generally, they were by far the most important factors to impact on our diverse survey respondent's ability to get involved in healthcare research and/or education, with time and caring responsibilities impacting on an individual's ability to get involved for women more than men.

As a time starved society, successful inclusivity of a diverse rich PPI community, will require offering a range of PPI opportunities (digital and non‐digital),^43^ adopting a blended flexible multilayered approach with varying degrees of time commitment involved. This will help to enable the most time deprived interested member of the public to see a way to getting involved and adding value. Whilst our survey results indicate hospital/university settings and local spaces as preferable locations to engage in PPI activity, it is important to note that these data were collected before the COVID 19 pandemic. Post the COVID 19 pandemic people are far more technologically savvy and are far more used to using platforms such as Teams and Zoom in both professional and social capacities, although there remains significant variability in access to such technology which much be considered when planning PPI.

Through our engagement events, we identified that most of the public do not understand what PPI in research and teaching is. Within the academic sector itself there are different terms to describe PPI activity. Within healthcare research and teaching in the UK, the term PPI has been universally adopted to mean research or teaching activity delivered with the public and the term ‘public engagement’ means telling/disseminating to the public including schools’ engagement. Outside of healthcare research and teaching, the term ‘public engagement’ is often used to encompass all these activities.^44^ This can cause problems when working across disciplines/groups. The term ‘participation’ is sometimes used within NHS settings to mean involvement which again can cause confusion in the healthcare setting, where individuals recruited to take part in research studies/clinical trials are called participants. It is important to recognise the use of different terms across university disciplines and relevant funders when trying to increase understanding within society about what PPI activity is. Before any sort of public communication strategy can be drafted, relevant funders, organisations; patients and publics, should agree on universal terminology that will facilitate the right messages being communicated to members of the public about how they can get involved in university research and teaching. In March 2022, this was achieved by a group of funders, regulators and research organisations coming together to sign a shared commitment to improve the extent and quality of PPI in health and social care research highlighting what PPI in health and social care research is.^45^

#### 4.1.3 The role of the PPI professional and support infrastructure

Jo Brett et al 2014,^46^ reported difficulties in incorporating PPI in meaningful ways due to researchers ‘lack of money and time’. To improve and increase the visibility and quality as well as the EDI aspects of PPI opportunities requires a level of recognition for the value of the PPI professional role in helping to facilitate PPI activity as part of a robust and efficient PPI support infrastructure. Mathie et al.^47^ recently put focus on the ‘invisible’ work of PPI leads. De Semoni et al.^48^ highlights that ‘this expertise should be valued as a fundamental part of research delivery and should be appropriately and adequately costed in grant bids. The long‐term benefit of properly costed PPI is sustainable, well‐supported PPI that has a greater likelihood of achieving impact’. Jinks et al.^49^ suggest that ‘sustaining PPI in research is a complex interplay of clarity of purpose, defined roles and relationships, organised support and a robust infrastructure that is well‐funded’.

Generally, PPI roles fall outside research and teaching support teams and are often fixed term and linked to specific grants. The existence of such roles is inconsistent across the sector. Investment in the creation and development of PPI professional teams and PPI support budgets as part of the core function and infrastructure within HEIs will enormously advance this work within the sector and ensure effective lay input.

Despite the plethora of PPI guidance available and the widespread recognition and support that PPI compliments and enhances both research and teaching activity in the delivery of training, grantmanship; evidencing research impact and obtaining and maintaining high levels of student satisfaction, our findings demonstrate that there is still a reticence for HEI's to invest or coordinate resources towards a dedicated core PPI support infrastructure. Further persuasion of the arguments is required to embed this activity, particularly in the current financially challenging environment the HE sector now finds itself in. Our findings highlight real and identified risks for failure to actively review strategy, policies and procedures to enable PPI activity to build and flourish within HE institutions, including: ‐ A lack of resource dedicated to the recruitment of target patient and public groups will compound issues of recruiting an inclusive and diverse public contributor population, to include a wide range of volunteer ages, clinical conditions and representation from underrepresented groups.

Now that it is universally accepted good practice for organisations to pay for time and cover costs associated with being involved in a PPI activity, for example, carer costs, institutional infrastructures need to develop timely nononerous flexible PPI payment processes and nonpayment incentive options (e.g., access to libraries and internal training courses; honorary titles). This approach, supported by our public survey results, would aim to provide all potential PPI contributors with a sense of reward for getting involved and recognise the individual needs of diverse members of society. For example, those who want to give their time voluntarily and those in receipt of benefits where the complications of accepting PPI/payment/vouchers is off putting and can act as a barrier.

Building on the learning from Greenhalgh et al. 2019^26^ that ‘a single, one‐size fits all framework may be less useful than a range of resources that can be adapted and combined in a locally generated co‐design activity’. The findings of this study affirm the careful balance required to support local research and teaching teams to build their own PPI frameworks^50^ alongside the necessity of host institutions to adopt supportive enabling infrastructures which go some way to enable PPI activity and not act as barriers in themselves. Working across any large institution there will be examples of existing PPI activity being embedded within a department and being done well and examples where it's not done so well. What this study has highlighted is that institutions with a strong healthcare research and teaching load need to play their part to embed PPI activity within their culture and support this activity at a wider university level, ensuring policies/procedures and infrastructure are inclusive and fit for purpose.

## 5 STRENGTHS AND LIMITATIONS

This study engaged a diverse section of the Cardiff population (drawing on the high footfall of visitors, patients, families, staff and students passing through two hospital concourse areas in the week between 10 AM and 4 PM as well as parents attending a weekday evening (6 PM–10 PM) family focused event in the local science discovery centre) to identify and address the barriers and enablers to support effective PPI within healthcare research and education. Cardiff is a rich multiculturally city, home to four HEIs (Cardiff University; Cardiff Metropolitan University of Wales; Royal Welsh College of Music and Drama). Focus group work also included professional service staff within Cardiff University who play a key role alongside academic staff to deliver a quality PPI experience for contributors who get involved in university research and teaching.

A limitation of this study is that despite two survey locations being on hospital sites that draw in populations from a wider geographical area, it would be good to review responses from other areas of Wales that are not home to a HEI. It would also be helpful to conduct the survey in different high footfall locations such as shopping centres to grow the representative and diverse sample. Another limitation is that the focus groups were run within one university setting based in Wales. It would be valuable to explore comparable responses from the three identified stakeholder groups across several HEIs in Wales, operating within the framework of the Wales devolved government. It is acknowledged that the number of focus group participants (four in each) is small. A final limitation of the study relates to the demographic options that were included in the survey. A broader range of variables should be included in future surveys, including, for example, gender identity, marital status, family income, and education. Future surveys with larger sample sizes should aim to conduct subgroup analysis focused on whether PPI barriers and enablers vary across demographic groups.

## 6 CONCLUSION

This study has identified that a diverse population are keen to get involved in university healthcare research and education, presenting an opportunity to build on public willingness to get involved. This will, however, require HEIs to be proactive in investing and developing an inclusive PPI infrastructure to support the culture change required to bring about consistent, high‐quality diverse PPI in health research and education and, ultimately, the shared responsibility for health vision.^51^

## AUTHOR CONTRIBUTIONS

**Sarah Hatch**: Conceptualisation; funding acquisition; visualisation; methodology; project administration; resources; data collection; formal analysis; writing—original draft; writing—review and editing. **Jim Fitzgibbon**: Writing—original draft; writing—review and editing. **Amanda Jayne Tonks**: Conceptualisation; funding acquisition; methodology; project administration; data collection; formal analysis; writing—original draft; writing—review and editing. **Liz Forty**: Conceptualisation; funding acquisition; methodology; project administration; data collection; formal analysis; writing—original draft; writing—review and editing.

## CONFLICT OF INTEREST STATEMENT

The authors declare no conflict of interest.

## ETHICS STATEMENT

The study was reviewed and approved by the local ethical approval committee at Cardiff University School of Medicine.

## TRANSPARENCY DECLARATION

S. H. affirms that the manuscript is an honest, accurate and transparent account of the study being reported; that no important aspects of the project have been omitted; and that any discrepancies from the project as planned (and, if relevant) have been explained.

## Supporting information

Supporting information.

## Data Availability

The data that support the findings of this study are available from the corresponding author upon reasonable request.
