# Supplementary material for: Diversity in patient and public involvement in healthcare research and education—Realising the potential
Source: Health Expect. 2023 Oct 23;27(1):e13896. doi: 10.1111/hex.13896 (PMC10726264; doi:10.1111/hex.13896)
Supplement: Supplementary file 1 — Supporting information. [file HEX-27-e13896-s001.docx]

**Appendix 1: Public/Patient Inclusive Questionnaire**

1. **Would you be interested in becoming involved in healthcare research and /or education at Cardiff University?**

**Please circle your answer: Yes / No / Already involved**

1. **If you answered yes, which of the following activities would you be interested in (please tick any that apply)?**

□ Sharing personal and family experiences of healthcare

□ Direct delivery of teaching to students

□ Curriculum or course planning

□ Recruitment and selection of students

□ Recruitment and selection of staff

□ Practice learning (in a clinical area)

□ Student assessment

□ Course evaluation and / or course management

□ Involvement in research management group meetings

□ Involvement in research advisory groups

□ Involvement in management meetings

□ Other (please state): _________________________________________________

1. **If no, would you be prepared to share some reasons why you answered in this way?**

______________________________________________________________________________________________________________________________________________________________

1. **If already involved, please state how you are involved.**

______________________________________________________________________________________________________________________________________________________________

1. **Where would you prefer to carry out research or education activities?**

□ On the university campus

□ In a local hospital

□ In another healthcare setting, e.g. doctor’s surgery

□ In a local space, e.g. community centre

□ From home via Skype / video conferencing / online?

□ Other (please state): __________________________________________

1. **Are any of the following factors relevant to your ability to become involved in healthcare research and / or education?**

□ Finding out / being aware of opportunities

□ Financial issues

□ Accessibility (e.g. transport / travel to locations, language)

□ Availability of training and support

□ Caring Responsibilities

□ Availability of time

□ Do not feel very confident

□ Not really sure about what I could contribute

□ Other (please state): ____________________________________________

**Would you be willing to be contacted by our team at Cardiff University for further opportunities to get involved? Yes / No**

If yes, please can you provide your preferred contact details:

Address:

Email

Telephone:

Thank you for your time. For further information please contact Mrs. Sarah Hatch, Engagement Manager, via medicengagement@cardiff.ac.uk or 02920 746735.

**A bit about you (please leave blank anything that you would prefer not to complete).**

| **Nationality -** |
| --- |
|  |

| **Sex -** Which of the following describes how you think of yourself? | |
| --- | --- |
| Male: |  |
| Female: |  |
| In another way: |  |
| Prefer not to say: |  |

| **Sexual Orientation -** What is your sexual orientation? | |
| --- | --- |
| Bisexual: |  |
| Gay man: |  |
| Gay woman/lesbian: |  |
| Heterosexual/straight: |  |
| Other: |  |
| Prefer not to say: |  |

| **Age -** | |
| --- | --- |
| 18-24 |  |
| 25-44 |  |
| 45-64 |  |
| 65-74 |  |
| 75+ |  |
| Prefer not to say |  |

| **Ethnicity -** What is your ethnic origin? | |
| --- | --- |
| White: |  |
| Gypsy or Traveller: |  |
| Black or Black British – Caribbean: |  |
| Black or Black British – African: |  |
| Prefer not to say: |  |
| Other Black background: |  |
| Asian or Asian British – Indian: |  |
| Asian or Asian British – Pakistani: |  |
| Asian or Asian British – Bangladeshi: |  |
| Chinese: |  |
| Other Asian background: |  |
| Mixed - White and Black Caribbean: |  |
| Mixed - White and Black African: |  |
| Mixed - White and Asian: |  |
| Other mixed background: |  |
| Arab: |  |
| Other ethnic background: |  |
| Not known: |  |
| Prefer not to say: |  |

| **Religion & Belief -** What is your religion /belief? | |
| --- | --- |
| No religion: |  |
| Buddhist: |  |
| Christian: |  |
| Hindu: |  |
| Jewish: |  |
| Muslim: |  |
| Sikh: |  |
| Spiritual: |  |
| Any other religion or belief: |  |
| Prefer not to say: |  |

| **Disability -** Do you consider yourself to be disabled? |
| --- |

| **If you have answered ‘Yes’, please tell us which of these best describes your disability:** | |
| --- | --- |
| Specific learning disability (such as dyslexia or dyspraxia): |  |
| General learning disability (such as Down's syndrome): |  |
| Cognitive impairment (such as autistic spectrum disorder or resulting from head injury): |  |
| Long-standing illness or health condition (such as cancer, HIV, diabetes, chronic heart disease, or epilepsy): |  |
| Mental health condition (such as depression or schizophrenia): |  |
| Physical impairment or mobility issues (such as difficulty using arms or using a wheelchair or crutches): |  |
| Deaf or serious hearing impairment: |  |
| Blind or serious visual impairment: |  |
| Other type of disability: |  |
| Prefer not to say: |  |

| **If "Other" is selected please provide a brief description of the nature of your disability as best you can:** |
| --- |
|  |
